# HDAC pharmacological inhibition promotes cell death through the eIF2α kinases PKR and GCN2

**DOI:** 10.18632/aging.100216

**Published:** 2010-10-31

**Authors:** Philippos Peidis, Andreas I. Papadakis, Kamindla Rajesh, Antonis E. Koromilas

**Affiliations:** ^1^ Lady Davis Institute for Medical Research, Sir Mortimer B. Davis-Jewish General Hospital, Montreal, Quebec H3T 1E2, Canada; ^2^ Division of Experimental Medicine, Faculty of Medicine, McGill University, Montreal, Quebec H3A 1A3, Canada; ^3^ Department of Oncology, Faculty of Medicine, McGill University, Montreal, Quebec H2W 1S6, Canada

**Keywords:** eIF2α phosphorylation, vorinostat, PKR, GCN2, apoptosis, HDACi

## Abstract

Histone deacetylase inhibitors (HDACi) comprise a family of chemotherapeutic agents used in the clinic to treat cutaneous T-cell lymphoma and tested for the therapy of other malignancies. Previous reports have shown that eIF2α phosphorylation is induced upon treatment with HDACi. However the kinase responsible for this phosphorylation or the biological significance of this finding is not yet established. Herein, we show that eIF2α phosphorylation is not attributed to a specific eIF2α kinase, but rather different eIF2α kinases contribute to its upregulation in response to the HDACi, vorinostat. More importantly our data indicate that eIF2α phosphorylation acts in a cytoprotective manner, whereas the eIF2α kinases PKR and GCN2 promote vorinostat-induced apoptosis. These results reveal a dual nature for eIF2α kinases with potential implications in the treatment with histone deacetylase inhibitors.

## INTRODUCTION

Histone deacetylase inhibitors (HDACi) represent an emerging class of anticancer treatments. The most prominent one, suberoylanilide hydroxamic acid (SAHA, vorinostat, Zolinza) was the first one to be approved by Food and Drug Administration (FDA) for the treatment of cutaneous T-cell lymphoma [1], followed by romidepsin [2], and others like LBH589 and valproic acid are in clinical trials [3]. Apart from their autonomous antitumor capacity, these drugs have been reported to be synergistic with a number of commonly-used anticancer agents, further signifying their potential as therapeutic agents [4].

HDACi act partially by inhibiting histone deacetylases, thus inducing the acetylation of histones. This modification renders chromatin in an active “open” state that allows accessibility to the transcriptional machinery [5]. Although the reversal of aberrant epigenetic states is thought to be the primary mode of action for these drugs, it has been demonstrated that these agents lead to acetylation of many non-histone factors including transcription factors, chaperones, DNA repair proteins and structural proteins [6]. These novel HDACi substrates which are involved in a wide range of signaling pathways and regulate a diverse array of biological processes, also partially contribute to the anticancer effects of these drugs.

One of the signaling pathways known to be activated by multiple stimuli is the one that converges on eIF2α phosphorylation [7]. This modification renders eIF2α from a substrate of the guanine-nucleotide exchange factor, eIF2B, to its inhibitor [8], which subsequently leads to an inhibition of mRNA translation. The general “shutdown” of protein synthesis provides the cell an opportunity to respond to the stress, and can act either by facilitating or protecting from cell death. This decision on cell fate mainly depends on the type and duration of the stress [9;10].

The kinases that phosphorylate eIF2α belong to a family of four members and are activated by different kind of stimuli; the interferon-inducible protein kinase R (PKR) in response to dsRNA, the endoplasmic reticulum (ER)-resident kinase (PERK) by accumulation of unfolded proteins in the ER, the general amino acid nonderepressing kinase 2 (GCN2) in response to aminoacid starvation, and the heme-regulated inhibitor (HRI) by low levels of heme or iron [8]. While it is believed that the kinases act mainly by modulation of the translation machinery, other functions unrelated to this specific mode of action have been described for PKR, PERK and GCN2 [11-13].

The contribution of the eIF2α phosphorylation pathway in the response to HDACi has not been thoroughly explored. In the few examples present in the literature, it has been demonstrated that treatment of the eosinonophilic leukemic cell line Eol-1 with HDACi leads to differentiation of these cells to mature eosinophils. This transformation to a differentiated state was attributed to a downregulation of the oncogenic fusion protein Fip1-like1-platelet-derived growth factor receptor alpha (FIP1L1-PDGFRA), whose mRNA translation was blocked by an induction in eIF2α phosphorylation [14-16]. In a recent study, PERK was shown to promote apoptosis in response to vorinostat in the U251 glioblastoma cell line through undefined mechanisms [17]. Moreover eIF2α phosphorylation was induced in response to paraborinostat in MCF-7 breast cancer and in JeKo-1 mantle cell lymphoma cell line [18;19].

In this report we show that eIF2α phosphorylation is not only induced in response to vorinostat treatment but also protects against the cytotoxic effects of this agent. Furthermore, this modification is a combinatorial event, involving more than one of the eIF2α kinases. More interestingly, PKR and GCN2 were found to enhance the sensitivity of the cells to this HDACi, thus opposing the cytoprotective role of eIF2α phosphorylation.

## RESULTS

### eIF2α phosphorylation is induced in human cancer and mouse cells upon treatment with vorinostat

**Figure 1.**
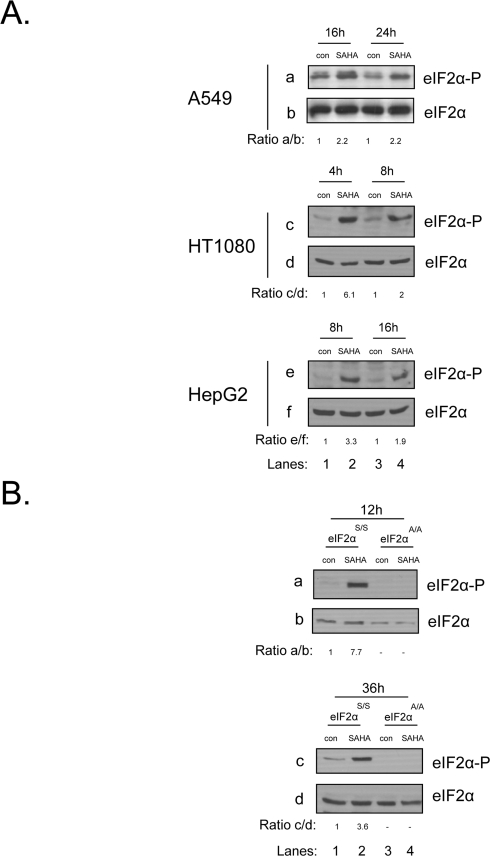
eIF2αphosphorylation is induced in human cancer and mouse cells upon treatment with vorinostat. (**A**) A549, HT1080 and HepG2 cells were treated with DMSO (con) or 10 μM of vorinostat (SAHA) for the indicated time periods. Protein extracts (50 μg) were subjected to western blot analysis for phosphorylated eIF2α (*panels* a, c, e) and total eIF2α (*panels* b, d, f). (**B**) eIF2α^S/S^ and eIF2α^A/A^ MEFs were treated with DMSO (con) or 10 μM of vorinostat (SAHA) for the indicated time periods. Protein extracts (50 μg) were subjected to western blot analysis for phosphorylated eIF2α (*panel* a) and total eIF2α (*panel* b). Representative blots are shown. The ratio of the phosphorylated protein to total normalized to its control is indicated. Quantification of the bands was performed by densitometry using the Scion Image software.

An earlier report has shown that in response to vorinostat treatment, eIF2α phosphorylation is induced in the U251 glioblastoma cell line [17]. We wished to examine if this upregulation is a general phenomenon met in human cancers. To this end we treated A549 lung carcinoma, HT1080 fibrosarcoma and HepG2 hepatocarcinoma cells with this drug and observed an increase in the levels of phosphorylated eIF2α at all time points examined (Figure 1A). To extend our study we also treated mouse embryonic fibroblasts (MEFs) with vorinostat, where we observed a similar upregulation in eIF2α phosphorylation. As control for eIF2α phosphorylation, we used MEFs that cannot be phosphorylated on Ser51, due to a mutation of Ser to Ala (eIF2α^A/A^), alongside their wildtype counterpart MEFs (eIF2α^S/S^) (Figure 1B). Taken together, we conclude that activation of the eIF2α phosphorylation pathway upon treatment with this agent is a common phenomenon observed both in human and mouse cells.

### Multiple eIF2α kinases are responsible for the induction of eIF2α phosphorylation upon treatment with vorinostat

**Figure 2.**
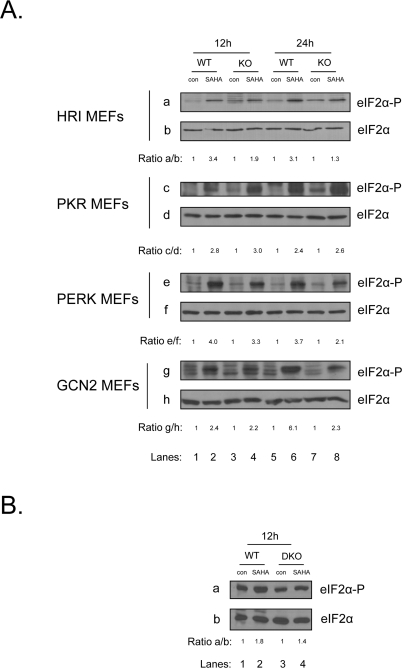
Multiple eIF2α kinases are responsible for the induction of eIF2α phosphorylation upon treatment with vorinostat. (**A**) The indicated MEFs were treated with DMSO (con) or 10 μM vorinostat for the indicated time periods. Protein extracts (50 μg) were subjected to western blot analysis for phosphorylated eIF2α (*panels* a, c, e, g) and total eIF2α (*panels* b, d, f, h). (**B**) GCN2 ^-/-^ PERK ^-/-^ MEFs (DKO) were treated together with their isogenic control (WT) with DMSO (con) or 10 μM vorinostat (SAHA) for the indicated time periods. Protein extracts (50 μg) were subjected to western blot analysis for phosphorylated eIF2α (*panel* a) and total eIF2α (*panel* b). Representative blots are shown. The ratio of the phosphorylated protein to total normalized to its control is indicated. Quantification of the bands was performed by densitometry using the Scion Image software.

Next we wished to determine which of the eIF2α kinases is responsible for mediating eIF2α phosphorylation in response to vorinostat. To this end, we treated MEFs deficient in each of the four eIF2α kinases together with their isogenic wildtype MEFs with the chemotherapeutic agent and examined eIF2α phosphorylation. Consistent with the previous findings, we detected an induction of eIF2α phosphorylation in all MEFs examined. However, even though the induction of eIF2α phosphorylation was lower in the knockouts (KO) of PERK, GCN2 and HRI compared to their isogenic wildtype cells (WT), it was not completely abolished in any of them, suggesting that vorinostat can activate more than one of the eIF2αkinases (Figure 2A). The redundancy of the eIF2α kinases was further confirmed by the use of double knock-outs of GCN2 and PERK(DKO) where the upregulation of eIF2α phosphorylation was only partially diminished in the absence of the two kinases (Figure 2B), further indicating that the induction observed, is a combinatorial event involving multiple kinases.

### eIF2α phosphorylation protects against vorinostat-induced cell death

**Figure 3.**
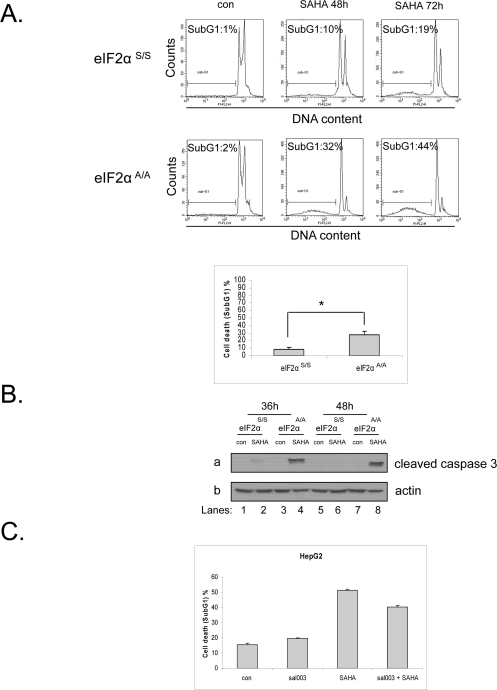
Phosphorylation of eIF2α protects against vorinostat-induced cell death. (**A**) eIF2α^S/S^ and eIF2α^A/A^ MEFs were treated with DMSO (con) or 10 μM vorinostat (SAHA) for 48h and 72h and were subjected to FACS analysis after propidium iodide staining. Cell death is represented by the percentage (%) of cells in SubG_1_. Histograms represent the mean cell death from three independent experiments for 48h of treatment (N=3, treated minus untreated). Bars denote S.E.M.. Statistical significance of the difference as calculated by *Student's t-test* is with *P<0.02. (**B**) The indicated MEFs were treated with DMSO (con) or 10 μM vorinostat for the indicated time periods. Protein extracts (50 μg) were subjected to western blot analysis for cleaved caspase 3 (*panel* a) and actin (*panel* b). A representative blot is shown. (**C**) HepG2 cells were treated with DMSO (con), 20 μΜ sal003, 10 μM vorinostat (SAHA) or both drugs for 24h and subjected to FACS analysis after propidium iodide staining. Cell death is represented by the percentage (%) of cells in SubG_1_. Histograms represent the mean cell death from three independent experiments for 24h of treatment (N=3). Bars denote S.E.M.. Statistical significance of the group difference as calculated by *ANOVA* is with *P<0.0001.

It is established in the literature that eIF2α phosphorylation can play both cytoprotective or proapoptotic roles depending on the type and duration of stress [10;20]. Here, we wished to investigate the effect of eIF2α phosphorylation in respect to cell fate upon treatment with vorinostat. To this end, we treated eIF2α^S/S^ and eIF2α^Α/Α^ MEFs with this drug and measured the cell death index by FACS analysis using propidium iodide (PI) staining. Our data show that eIF2α^Α/Α^ MEFs are more sensitive to this drug than eIF2α^S/S^ MEFs, indicating that eIF2α phosphorylation protects against vorinostat-induced cell death (Figure 3A). In order to confirm the FACS analysis data we examined the levels of cleaved caspase 3, a downstream effector of apoptosis. We observed high levels of cleaved caspase 3 in the treated eIF2α^Α/Α^ MEFs, in contrast to the treated eIF2α^S/S^ MEFs where cleaved caspase 3 was almost not detectable (Figure 3B). To extend our observations to human cells, we treated HepG2 cells with vorinostat together with a derivative of salubrinal [21], sal003, a compound that increases phosphorylation of eIF2α by blocking its dephosphorylation. Treatment with both agents decreased the cell death index in the co-treated cells compared to the cells treated only with the HDACi (Figure 3C), further validating that eIF2α phosphorylation protects from the apoptotic effects of the drug not only in mouse but also in human cells.

### eIF2α kinases enhance sensitivity to vorinostat independently of eIF2α phosphorylation

**Figure 4.**
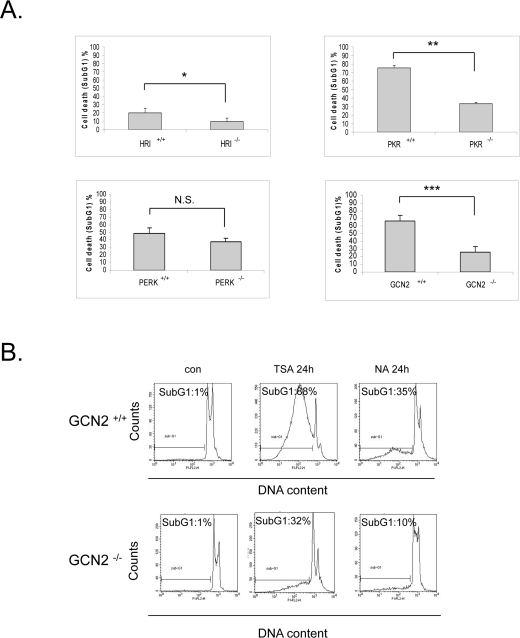
eIF2α kinases enhance sensitivity to vorinostat independently of eIF2α phosphorylation. **(A)** The indicated MEFs were treated with DMSO (con) or 10 μM vorinostat (SAHA) for the different time periods (HRI MEFs 72h, PKR and PERK MEFs 48h, GCN2 MEFs 24h). Cells were subjected to FACS analysis after propidium iodide staining. Cell death is represented by the percentage (%) of cells in SubG_1_. Histograms represent the mean cell death from three independent experiments for the corresponding time periods (N=3, treated minus untreated). Bars denote S.E.M.. Statistical significance of the differences as calculated by *Student's t-test* is with *P<0.03 **P<0.004, ***P<0.005. N.S. = not significant. **(B)** GCN2 ^+/+^ and GCN2 ^-/-^ MEFs treated with DMSO (con), 1 μM trichostatin A (TSA) or 50 mM nicotinamide (NA) for 24h. Cells were subjected to FACS analysis after propidium iodide staining. Cell death is represented by the percentage (%) of cells in SubG_1_.

We have previously demonstrated that the eIF2α kinase PKR can promote doxorubicin-induced apoptosis in an eIF2α independent manner [22]. In order to examine if the biological effect of the eIF2α kinases in response to vorinostat is mediated by eIF2α phosphorylation or is independent of it, we treated wildtype and knockout MEFs of each of the four kinases with this drug and subjected them to FACS analysis. In contrast to the observation that eIF2α phosphorylation was cytoprotective, GCN2 and PKR significantly increased the sensitivity of the cells to the HDACi (about 40% in both cases) as indicated by the differences in the cell death index between the wildtype and knockout MEFs. A similar trend but to a much lesser extent was observed in HRI^+/+^ and HRI ^-/-^ MEFs. On the contrary no significant differences in SAHA induced-cytotoxicity were observed in PERK ^+/+^and PERK ^-/-^ MEFs (Figure 4A).

Further we wished to investigate whether this proapoptotic effect is specific to vorinostat or if it takes place with other HDACi. To this end, we treated GCN2 ^+/+^and GCN2^-/-^ MEFs with trichostatin A (TSA), a structurally and functionally similar HDACi to vorinostat, and nicotinamide, a molecule that targets HDAC family members that are not inhibited by vorinostat or TSA. Similar to what we observed for vorinostat, these two compounds were more cytotoxic in the presence of GCN2 (Figure 4B). Taken together, these data support that in response to HDAC inhibition the eIF2α kinases mediate a proapoptotic role independently of eIF2α phosphorylation.

## DISCUSSION

In this study we report that eIF2α phosphorylation is increased upon treatment with vorinostat and that this induction is a common phenomenon observed both in human and mouse cell lines. While the biological effect of this modification is to enhance the resistance of cells to this agent, PKR and GCN2 confer an opposite phenotype, promoting vorinostat-induced cytotoxicity. Considering that certain cancers don't readily respond to HDAC inhibition and that some malignancies that initially respond to these treatments will develop resistance [23], it is essential to determine the factors that contribute to chemosensitivity and those which prevent chemotherapeutics from exerting their cytotoxic effects.

HDACi can promote cell death through a variety of ways [24]. Although the pathways that link eIF2α phosphorylation to the apoptotic program are well understood [7], little is known about how can eIF2α kinases activate cell death pathways independently of eIF2α phosphorylation. We have previously established that PKR can promote doxorubicin-induced apoptosis through JNK in a eIF2α independent fashion [22]. Moreover, PKR has been demonstrated to physically interact with Fas-associated protein with death domain (FADD) [25] and inhibitor of kappa B kinase (IKK) [26], the latter protein mediating the tumor necrosis factor (TNF) induced response [27], further linking this eIF2α kinase to the apoptotic pathway in a eIF2α independent manner.

An important question that needs to be addressed is how the HDACi activate the eIF2α kinases. One possibility is that they do so by altering the interactions between eIF2α kinases with chaperones. It has been demon-strated that all of the eIF2α kinases are held in a dormant state through their interaction with chaperones and treatment with geldanamycin, an agent that changes the conformation of hsp90 chaperone, regulates the activity of PKR [28], GCN2 [29] and HRI [23]. To support this notion, HDACi have been shown to lead to the acetylation of heat shock protein 90 (hsp90), resulting in the subsequent release of its client poteins like the breakpoint cluster region-abelson fusion protein (Bcr-abl) [31], and acetylation of the ER-resident chaperone glucose-regulated protein 78 (GRP78), leading to activation of PERK [17]. Therefore it is highly plausible that acetylation of these chaperones by vorinostat orchestrates the simultaneous activation of all the eIF2α kinases in the cell. This is not the first report showing that anticancer agents lead to the parallel activation of multiple eIF2α kinases. Flavonoids, which intriguingly can also modulate hsp90 function and levels [32;33], have also been demonstrated to simultaneously activate HRI, PKR and PERK [34], further indicating the need to characterize the mechanisms of eIF2α kinase activation in response to chemotherapeutics.

In conclusion this study unveils the contribution of the eIF2α phosphorylation pathway in the biological outcome to vorinostat treatment. We have demonstrated the involvement of eIF2α phosphorylation as a cytoprotective mechanism against this agent. In contrast, we show that the eIF2α kinases display divergent roles which oppose the cytoprotective effect of eIF2α phosphorylation. Although GCN2 expression has not been documented so far to be increased in any malignancy, PKR levels and/or activity have been reported to be elevated in breast cancer [35], melanoma and colon cancer cell lines [36] and hematological malignancies [37], raising the possibility that these cancers might be good candidates for treatment with these agents. The proapoptotic effects of these kinases to HDAC inhibition could be further augmented in cancers where eIF2α phosphorylation effects on translation are counteracted by overexpression of eIF2Β[38]. Furthermore HDACi could be used in combination with strategies that block this phosphorylation such as expression of eIF2α mimetic proteins like the hepatitis C virus (HCV) E2 protein [39] or proteins that induce eIF2α dephosphorylation like human papillomavirus (HPV) E6 protein [40]. As such the eIF2α phosphorylation pathway could be exploited to improve the treatment with HDACi.

## METHODS

### Cell culture and treatments

MEFs deficient in each of the four eIF2α kinases or deficient in eIF2αphosphorylation were maintained as previously described [11]. GCN2 ^-/-^ PERK ^-/-^ MEFs and their wildtype counter-parts were cultured as previously described [41]. HT1080 and A549 cells were grown as previously described [10]. HepG2 cells were maintained as previously described [42]. Cells were treated with 10 μM of vorinostat (ChemieTek, Indianapolis, IN, USA) dissolved in DMSO, 20 μΜ of sal003 (ChemBridge, San Diego, CA, USA) dissolved in DMSO, 1 μM trichostatin A (Sigma, Oakville, ON, Canada) dissolved in DMSO or 50 mM nicotinamide (Sigma) dissolved in H_2_O. The controls were treated with an equal amount of the solvent.

### Cell staining and flow cytometry analysis

FACS analysis using propidium iodine staining was performed as previously described [40].

### Protein extraction and immunoblotting

Extraction of proteins from cells and western blot analysis were performed as previously described [40]. For immunoblotting of cleaved caspase 3 extracts were prepared as previously described [43]. The antibodies used in this study are previously described in [22].

### Statistical analysis

All quantitative variables are presented as means ± S.E.M. We compared the differences of more than two groups using one-way ANOVA and the differences of two groups using two-tailed Student *t* test (GraphPad Prism 5, La Jolla, CA, USA), and P<0.05 was considered statistically significant.

## Abbreviations

Bcr-abl: breakpoint cluster region-abelson
DMSO: dimethyl sulphoxide
dsRNA: double-stranded RNA
eIF2α: eukaryotic initiation factor 2 subunit alpha
eIF2B: eukaryotic initiation factor 2B
ER: endoplasmic reticulum
FACS: fluorescence-activated cell sorting
FADD: Fas-associated protein with death domain
FDA: food and drug administration
FIP1L1-PDGFRA: Fip1-like1-platelet-derived growth factor receptor alpha
GCN2: general control non-derepressible 2
hsp90: heat shock protein 90
GRP78: glucose-regulated protein 78
HCV: hepatitis C virus
HDACi: histone deacetylase inhibitors
HPV: human papillomavirus
HRI: heme-regulated inhibitor
IKK: inhibitor of kappa B kinase
JNK: c-jun NH_2_-terminal kinase
MEF: mouse embryonic fibroblast
PERK: PKR-like endoplasmic reticulum-resident kinase
PI: propidium iodide
PKR: double-stranded RNA-dependent protein kinase
SAHA: suberoylanilide hydroxamic acid
TNF: tumor necrosis factor
TSA: trichostatin A
